# P_2_O_5_-Free Cerium Containing Glasses: Bioactivity and Cytocompatibility Evaluation

**DOI:** 10.3390/ma12193267

**Published:** 2019-10-08

**Authors:** Gigliola Lusvardi, Francesca Sgarbi Stabellini, Roberta Salvatori

**Affiliations:** 1 Department of Chemical and Geological Sciences, University of Modena and Reggio Emilia, Via Campi 183, 41125 Modena, Italy; sgarbistabellinifrancesca@gmail.com; 2 Lab. Biomaterials, Department of Medical and Surgical Sciences of Children & Adults, University of Modena and Reggio Emilia, Via Campi 213/A, 41125 Modena, Italy; roberta.salvatori@unimore.it

**Keywords:** bioactive glasses, cerium, bioactivity, cytocompatibility, cell proliferation

## Abstract

(1) Background: valuation of the bioactivity and cytocompatibility of P_2_O_5_-free and CeO_2_ doped glasses. (2) Methods: all glasses are based on the Kokubo (K) composition and prepared by a melting method. Doped glassed, K1.2, K3.6 and K5.3 contain 1.2, 3.6, and 5.3 mol% of CeO_2_. Bioactivity and cytotoxicity tests were carried out in simulated body fluid (SBF) solution and murine osteocyte (MLO-Y4) cell lines, respectively. Leaching of ions concentration in SBF was determined by inductively coupled plasma mass spectrometry (ICP-MS) and optical emission spectrometry (ICP-OES). The surface of the glasses were characterized by scanning electron microscopy (SEM) and X-ray diffraction (XRD) techniques. (3) Results: P_2_O_5_-free cerium doped glasses are proactive according to European directives. Cerium increases durability and retards, but does not inhibit, (Ca_10_(PO_4_)_6_(OH)_2_, HA) formation at higher cerium amounts (K3.6 and K5.3); however, cell proliferation increases with the amount of cerium especially evident for K5.3. (4) Conclusions: These results enforce the use of P_2_O_5_-free cerium doped bioactive glasses as a new class of biomaterials.

## 1. Introduction

Bioglasses are a class of biomaterials [1,2] widely used in bone defect regeneration for their ability to bond and integrate with soft and hard tissues in the living body. The first bioglass (45S5 Bioglass^®^) was developed by Hench in 1969 [3] for bone regeneration. Another important bioglass is the phosphate-free Kokubo glass, which shows comparable bioactivity concerning the 45S5 Bioglass^®^ [4]. These bioglasses were also modified by the addition of other components chosen to improve their properties and produce specific effects in the biological environment after their physiological release [5,6,7].

Our research focuses on the addition of therapeutic ions, such as zinc, fluoride, gold, copper, gallium, and cerium to these bioglasses [8,9,10,11,12,13,14,15,16]. The resulting glasses are bioactive and their behavior is comparable to Hench and Kokubo bioglasses. Their properties are also improved by functionalization with metallic nanoparticles and/or biomolecules and are adapted to the specific applications required (scaffolds, drug delivery systems, coatings) [17,18,19,20,21,22].

There has been renewed interest towards the use of rare earth elements, especially towards cerium and its catalytic properties [23,24,25,26,27,28]. Indeed, CeO_2_ nanoparticles [29,30,31,32,33] possess excellent catalytic activities deriving from quick interconversion between the Ce^4+^ and Ce^3+^ oxidation states; in the physiological environment, these nanoparticles have multi-enzymatic mimetic properties toward enzymes, such as superoxide dismutase (SOD) and catalase (CAT). Cerium can thus destroy reactive oxygen species (ROS) acting like the antioxidant species [34,35]. Cerium compounds are also particularly interesting for their pharmacological properties and are used as antiemetics, bacteriostatics, immunomodulatory agents, and antitumorals.

Our research has focused on the design of bioactive glasses with the ability to reduce oxidative stress after implantation, with the potential long-term clinical benefit of reducing the period of convalescence and the number of drugs needed for patient care.

To do so, we explored various glasses with different compositions, conditions of synthesis, and dimensions (powders of micro and meso dimensions or rectangular slices). We obtained glasses based on the 45S5 Bioglass^®^ and doped with an increasing amount of CeO_2_ (1.2, 3.6, and 5.3 mol%) [36]. After soaking in simulated body fluid (Dulbecco’ Modified Essential Medium, DMEM solution) these glasses, particularly the 1.2 and 3.6 mol% of CeO_2_ ones, are bioactive in terms of the formation of apatite (hydroxyapatite, HA). As preliminary cytocompatibility tests are required for in vivo applications [37,38], we treated them in a cell culture medium with a murine long bone osteocyte-like cell line (MLO-Y4); cerium-containing glasses show an increment in cell viability with respect to 45S5 Bioglass^®^; furthermore, no cell aggregation and deformation were observed at long incubation times (72 h). In particular, the glasses with 1.2 and 3.6 mol% of CeO_2_ are likely to be suited and are promising scaffolds for hard-tissue applications.

We have also demonstrated [28] that the cerium ions have a structural role in two series of cerium-containing glasses based on the Hench and Kokubo compositions and doped with 1.2, 3.6, and 5.3 mol% of CeO_2_. These studies indicated that, in all cases, the cerium ions are present in both oxidation states, Ce^3+^ and Ce^4+^, but while in the Kokubo-based glasses, cerium is coordinated by non-bridging oxygens (NBOs) originated from the disruption of the silicate network; in the P-containing Hench-based glasses, the NBOs around cerium ions belong to orthophosphate groups. The latter groups stabilize the Ce^3+^ species subtracting them from the interconversion process between Ce^3+^ and Ce^4+^, which is necessary for the catalase mimetic activity; conversely, Ce^4+^ is higher in the Kokubo-derived glasses showing higher bioactivity and higher catalase mimetic activity concerning Hench-derived glasses.

Finally, it is worth reminding that according to recent European directives, [29] it is necessary to evaluate the apatite-forming ability by validated methods, considering the effect of the size and superficial area and providing an adequate volume of simulated biological fluid to evaluate the bioactivity of bioactive glasses.

In this manuscript, we present the bioactivity and the cytocompatibility investigation of potential bioactive P_2_O_5_-free cerium-containing glasses based on the Kokubo composition, and modified by the addition of cerium oxide, to identify the best composition for the optimization of bioactivity, cytocompatibility, and catalase mimetic activity.

## 2. Materials and Methods

### 2.1. Synthesis of Glasses

The parent glass (hereafter named K) is the phosphate-free glass proposed by Kokubo et al. [4]; the molar compositions of the cerium-containing glasses (hereafter named K1.2, K3.6, and K5.3) are reported in Table 1.

The samples were prepared as reported in [28] by the melting method; for the in vitro tests, we used slices prepared as reported in [36].

### 2.2. In Vitro Apatite Formation Test

The in vitro HA formation ability of K, K1.2, K3.6, and K5.3 was verified by soaking glasses in SBF [39] at different times (1, 4, 7, 15, and 30 days) at 37 °C. The rectangular slices were washed with pure acetone and immersed in SBF to maintain a fixed volume to surface area ratio: V_SBF_ = 100·S_a,glass_. The dimensions of the slices are 3 mm of thickness and a surface of 1 cm^2^. These tests were performed in agreement with the European directives, which establish standard methods for obtaining a reproducible evaluation of bioactivity [40].

#### 2.2.1. Measurement of Element Concentrations

Glass dissolution in SBF was assessed by the evaluation of the changes in the concentration of silicon, calcium, sodium, phosphorus, and cerium; these solutions were measured by elemental analysis.

To detect silicon, calcium, sodium, and phosphorus, an Optima 5300 DV spectrometer (Perkin Elmer, Shelton, CT, USA) was used and to detect cerium an HR-MC-ICPMS Neptune mass spectrometer (Thermo Fisher Scientific Instrument, Bremen, Germany) was used. The concentrations are expressed as mean value by 5% and 1% of SD over the replicates, respectively, for Optima 5300 DV spectrometer and HR-MC-ICPMS Neptune mass spectrometer

#### 2.2.2. X-Ray Diffraction Analysis (XRD)

X-ray diffraction analysis was performed, after SBF soaking, to verify the formation of HA on the surface. The samples were analyzed in the (2θ) 5°–55° range, using X-ray diffraction apparatus (X’Pert PRO-PANAnalytical, Panalytical, Malvern, UK) equipped with Ni-filtered CuKα radiation (λ = 1.54060 Å).

### 2.3. Assessment of Cytocompatibility

The MLO-Y4 cell lines (Murine Long bone Osteocyte –Y4) were grown in DMEM (Euroclone, Milan, Italy) supplemented with 10% of fetal bovine serum (FBS) (Euroclone, Milan, Italy); incubators were placed in CO_2_ at 37 °C and used for the cytotoxicity test. The samples were used, both, for direct (neutral red absorption) and indirect (extract) contact for cytocompatibility tests (MTT and BrdU tests). To prepare the extract, the samples were immersed in DMEM (Invitrogen, Karlsruhe, Germany) without FBS, at 37 °C for 72 h. 1.25 cm^2^/mL was the ratio between the glass and DMEM as indicated by ISO 10993-5 [41]. After 72 h the extract was filtered using a 0.22 micron filter (Merck Millipore, Darmstadt, Germany) to eliminate possible microbial species and make the eluate suitable for incubation with the cells. After obtaining the extract from each sample, different sample dilutions (1:2, 1:5, 1:10) were tested to verify the cell’s viability at different concentrations.

#### 2.3.1. Direct Viability Test: Neutral Red Uptake (NR)

NR uptake (NR solution N2889 Sigma-Merck, Darmstadt, Germany) is a well-known parameter of cytotoxicity and is widely used to evaluate the number of viable cells in culture [42]. NR is a vital dye that accumulates in their lysosomes of viable cells. Cytotoxicity is calculated as a reduction of the NR uptake after 24 and 72 h of direct exposure to the material into the cells. Cells were seeded in a six multiwell and cultured at 37 °C ± 1 °C, 90% ± 5% humidity, and 5% ± 1% CO_2_/air for 24 and 72 h. 300 μL of NR solution was added, after removing the culture medium, to the well for 3 h. NR solution was discarded and cells rinsed with 300 μL of Dulbecco’s-Phosphate Buffer Solution (D-PBS, Euroclone, Italy). 1.5 mL of ethanol/acetic acid mixture was added to extract the dye from the cells. The quantity of extracted NR was measured using UV-visible spectrophotometry at 540 nm (Multiscan RC by Thermolab, ThermoFisher Scientific, Helsinki, Finland). All experiments were repeated three times for each sample and using DMEM without serum (CTRL-) and latex (CTRL+) as references.

#### 2.3.2. Indirect Viability Test: MTT

MTT is a rapid colorimetric test based on the cleavage of a yellow tetrazolium salt on purple formazan crystals by mitochondrial enzymes in metabolically active cells. It is used to evaluate indirect toxicity and cell viability by spectrophotometry. [43] MLO-Y4 cells were grown in 96 multiwell and placed in contact with the sample extract and their dilutions (1:2, 1:5, 1:10) for 24 and 72 h.

The sample extract was made in centrifuge tubes by adding DMEM and the sample at a ratio of 1.25 cm^2^/mL (according to ISO 10993-5 [41]). The tubes were incubated at 37 °C for 72 h and then filtered with a 0.22 micron filter for final use. At the end of the incubation period (24–72h), between the cells and the extract, MTT, tetrazolium salt (Cell Proliferation Kit I (MTT) Roche diagnostics, Indianapolis, IN, USA) was added to each well and waited for 2 h, to dissolve the formazan crystals formed DMSO (dimethylsulphoxide) was subsequently added to each well. The amount of formazan at 540 nm, using the UV-visible spectrophotometer, is directly associated with the activity of oxidoreductase-NAD (P) dependent enzymes in metabolically active cells.

#### 2.3.3. Proliferation Test: Bromo-2-deoxyUridine (BrdU)

BrdU test is a colorimetric immunoassay assay (Cell Proliferation ELISA, BrdU, and Roche) used to evaluate DNA synthesis [44]. BrdU is an analog of thymidine and it can be incorporated into DNA during S-phase [45]. It can be detected with an anti-BrdU specific antibody; the binding of the antibody requires the denaturation of the DNA. The BrdU labeling solution was added in cells grown in 96-well plates; after 24 and 72 h the proliferation extracts were evaluated by absorbance at 370 nm using the UV-visible spectrophotometer reported above. The absorbance related to the color developed is proportional to the amount of newly synthesized DNA.

#### 2.3.4. Statistical Analysis

Data from cytotoxicity tests were analyzed by a one-way analysis of variance (ANOVA) in a combination with Dunnett post hoc tests for data evaluation. The statistical test compares the means between the groups treated with samples and negative references; p ≤ 0.05 was considered significant. Statistical analysis was performed using GraphPad Prism 8.1 software (GraphPad Software, San Diego, CA, USA).

#### 2.3.5. Cell Morphology Observation

After incubation (24 and 72 h) and before the NR uptake test, the cell morphology was evaluated by optical microscopy (Leitz, Germania). Cell observation includes the evaluation of the presence of intracytoplasmatic granules, cell lysis, reduction of cell growth, all morphological parameters, which indicate a change from the normal morphology of cells. The adhesion of the cells to the surface of glasses was also verified using scanning electron microscopy by a FEI Quanta 200 Instrument (Fei Company, Eindhoven, The Netherlands), equipped with an INCA 350 EDS apparatus (Oxford Instruments, Abingdon, UK).

## 3. Results

**Figure 1:**
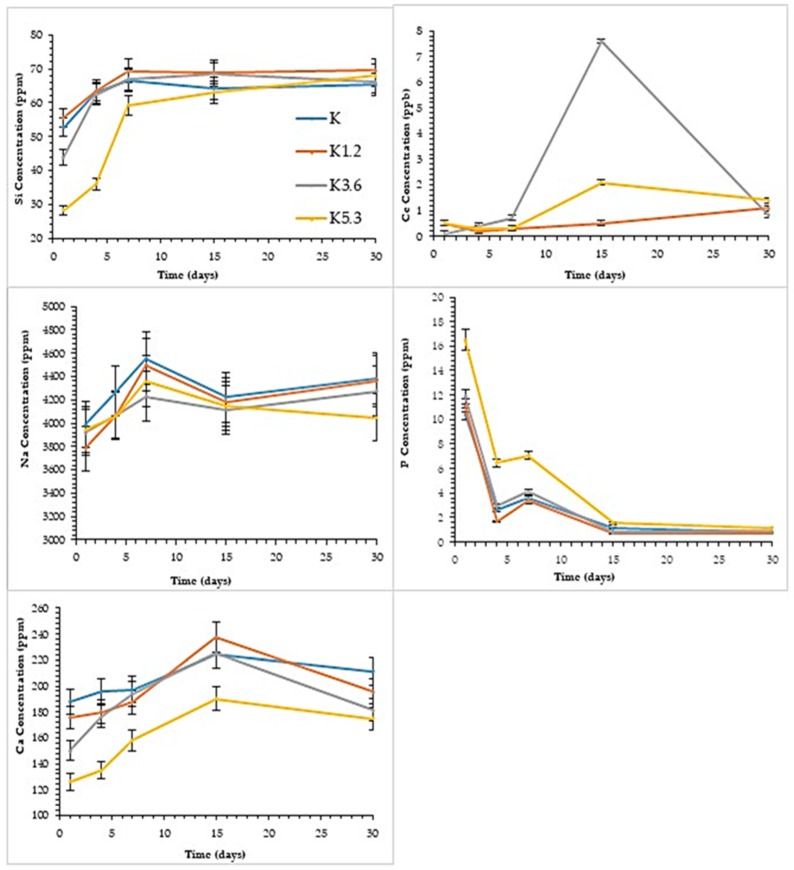
Ion concentrations in the simulated body fluid (SBF) solution vs. soaking time of the glasses.

The results from the elemental analysis deriving from the leaching of the glasses in SBF solution are summarized in Figure 1. Ce and Si are not present in the SBF starting solution and, therefore, the trend of the concentrations highlighted in the graphics depends essentially on the degree of glass solubilization. In the case of Ce, it is possible to observe an increase of the concentrations after 15 days for all glasses; between 15 and 30 days, the increase continues for K1.2 glass, whereas K3.6 and K5.3 glasses show a strong decrease. This is due to the precipitation of a calcium or calcium/cerium phosphate to the surface, as successively indicated by mineralogical analysis. The increase of the durability in the amount of Ce, as previously discussed [8], is here especially evident for the K3.6 and K5.3. After 30 days, all samples converge to a constant value of about 1–1.5 ppb.

For all glasses, Si concentrations increase progressively already from the first days of contact and converge to a constant value (about 70 ppm) in agreement with the gradual formation of a silica gel on the surface of the glasses [46]. Notice that after 4 and 7 days the K, K1.2, and K3.6 glasses released a higher amount of silicon than K5.3, which is a more durable glass.

As reported in our previous studies [8], we have considered the variation of Na concentration as the most significant for evaluating the durability of the glasses, as it is not involved in the precipitation of poorly soluble compounds. Na concentration increases for all glasses during the time, and lower amounts are related to the increasing amount of Ce. Some fluctuations are attributable to pH changes and the different ion exchange in the SBF solution.

For all glasses, Ca concentration increases towards 15 days and tends to decrease after 30 days: this is related to the formation of calcium or calcium/cerium phosphate on the surface, as reported in the case of Ce ions behavior. The lower concentration trend of K5.3 agrees with its higher durability.

Similarly, for all glasses, P concentration tends to decrease until reaching a constant value after 30 days, in agreement with the precipitation of the phosphatic phases. K5.3 shows higher P concentration, especially evident in 1, 4, and 7 days. All results are in line with a higher reticulation of the structure of the cerium doped glasses, which induces greater durability and slow release of cations that can precipitate with P.

The mineralogical analysis was performed on different glasses after SBF tests to verify the formation of apatite on the surfaces. By matching the JCPDS files [47], some peaks have been identified as the most intense peaks of Ca_10_(PO_4_)_6_(OH)_2_ [HA], which is indicative of the bioactivity of the glasses.

**Figure 2:**
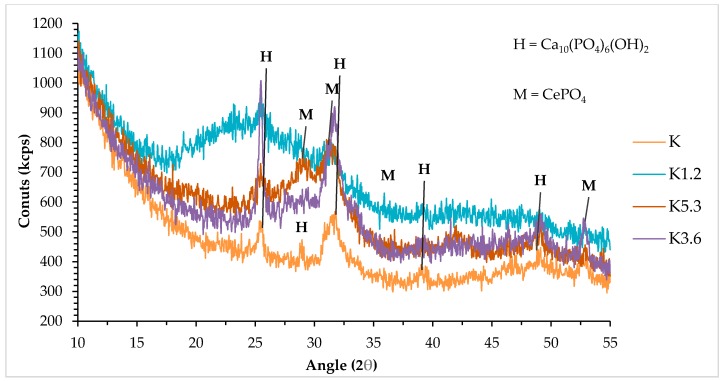
X-ray diffraction (XRD) patterns of the glasses, after 15 days of SBF soaking.

The HA formation has already occurred after 4 days (data not shown) and is more evident for K and K1.2 glasses. After longer times (15 days, Figure 2) and for K3.6 and K5.3 glasses, in addition to the presence of HA, another phosphatic phase was detected: CePO_4_ (Monazite). This in agreement with our previous results [8], the presence of cerium retards but does not inhibit the HA formation.

**Figure 3:**
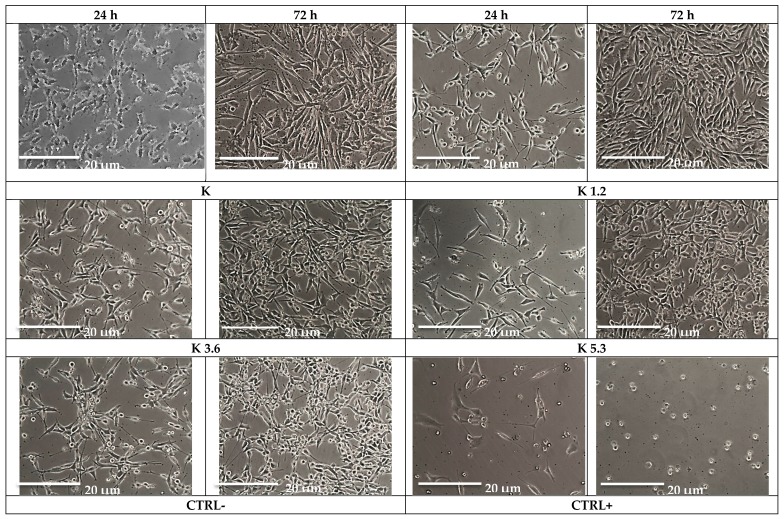
Cellular morphology for the glasses, negative control (CTRL–) and positive control (CTRL+) after 24 and 72 h.

Cytotoxicity tests of all samples were performed using the MLO-Y4 cell line because of the established and recognized efficacy of this cell line for in vitro studies involving materials for bone regeneration. Images (Figure 3) acquired with optical microscopy allow us to evaluate the cellular morphology after 24 and 72 h when cells are in contact with the glass. These images show excellent cellular viability, especially after 72 h, and a prominent proliferative activity. Figure 3 shows MLO-Y4 cells without morphological damage and intracytoplasmic vacuoles after contact with Ce-containing glasses; particulate residue observed with optical microscopy did not modify cellular morphology.

**Figure 4:**
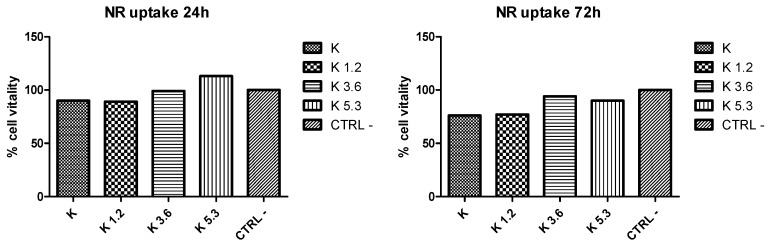
Neutral red (NR) uptake tests of murine osteocyte-like cell (MLO-Y4) cultures exposed to the glasses after 24 and 72 h.

NR uptake test (Figure 4) is used to evaluate the number of viable cells in culture; it is a quantitative evaluation of neutral red dye incorporated in cells obtained by optical density scanning. Both optical microscopy and NR uptake tests highlighted the excellent cellular viability. Of note, the viability of the cells increases with the increasing of cerium, especially evident after 24 h. After 72 h, the cellular vitality tends to decrease; however, the contact with K3 and K5, again, gives rise to higher values with respect to K and K1.2.

**Figure 5:**
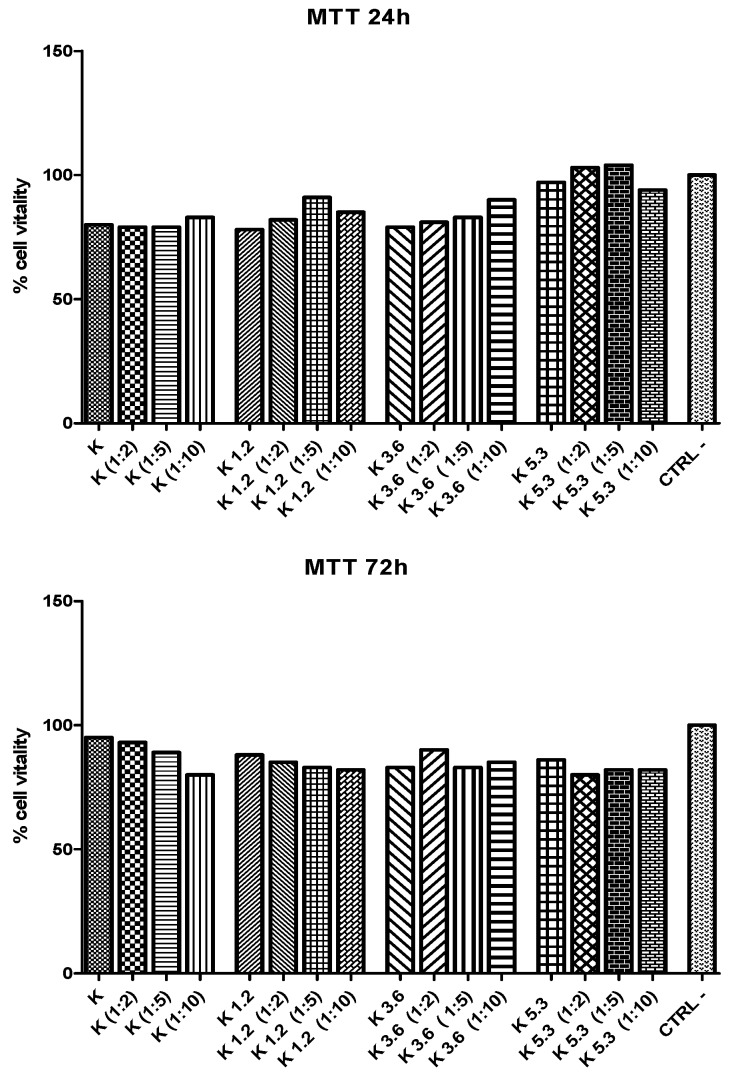
MTT tests of murine osteocyte-like cells (MLO-Y4) cultures exposed to extracts of the glasses and dilutions (1:2, 1:5, 1:10) for 24 and 72h.

MTT test (Figure 5) is used to evaluate indirect toxicity and the number of metabolic-active cells. A quantitative evaluation of yellow tetrazolium salt incorporated in cells was obtained by optical density scanning. Results show that the cellular vitality for K5.3 is higher than the one of other glasses. The cell viability of K5.3 increases also with dilution (particularly 1:2 and 1:5) after 24 h: this shows that the low-concentration of eluate from K5.3 has already a good effect on cellular vitality. These results show that the addition of cerium has a positive effect on the extra and intracellular environment that interacts with cellular stress defense mechanisms [48,49].

**Figure 6:**
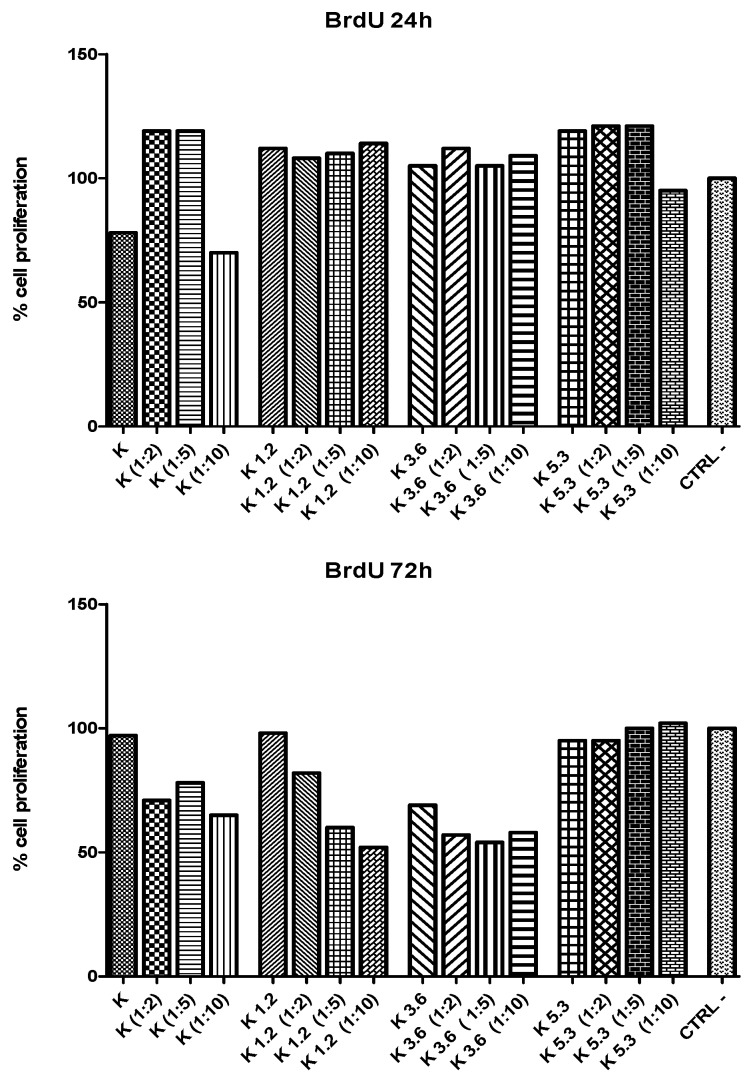
Cell proliferation (BrdU test) of murine osteocyte-like cells (MLO-Y4) cultures exposed to extracts of the glasses and dilutions (1:2, 1:5, 1:10) for 24 and 72 h.

The proliferation of cells cultured in eluates (and their dilutions) was further investigated using BrdU assay (Figure 6). A quantitative evaluation of BrdU incorporated into the newly-synthetized DNA of cycling cells was obtained by optical density scanning. After 24 h, there is a good cellular proliferation for all glasses and dilutions. After 72 h, cell proliferation decreases for diluted eluates of K, K1.2, and K3.6 glasses; conversely, K5.3 glass shows an excellent cellular proliferation that increases with the dilution also after 72 h.

Statistical analyses (Table 2 and Table 3) on the cytotoxicity tests were conducted with one-way variance analysis and Dunnet post hoc test; in all cases, the data are significant with a p < 0.05. The subsequent Dunnet post hoc test was performed only on samples without dilutions; the obtained value of R^2^ (coefficient of determination) indicates the correlation between the dependent (CTRL–) independent (K, K1.2, K3.6, K5.3) variables. The results show that all samples have a significant positive effect on cellular vitality in direct contact; while in indirect contact they indicate a variable effect. The effect of the variation of R^2^ is expressed in the comparison between (CTRL– and K3.6) and (CTRL– and K5.3) confirming that the increase in the amount of cerium added in glasses has a positive effect on the vitality and proliferation of the MLO-Y4 line.

**Figure 7:**
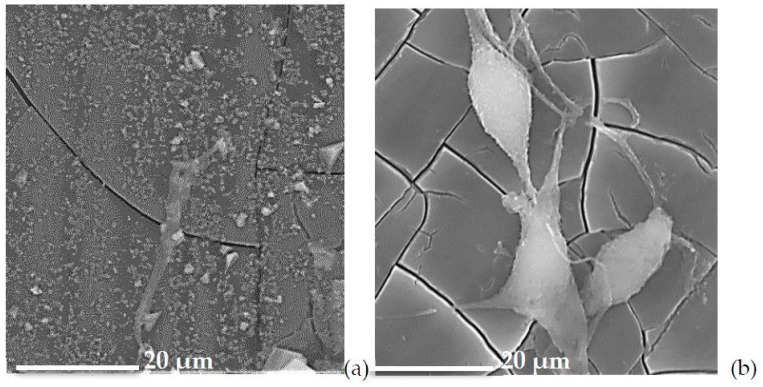
Micrographs of MLY04 cells adherent to the surface of K1.2 (**a**) and K 5.3 (**b**) glasses.

The morphological evaluation (Figure 7) underlines the difference between the glasses with the lowest (K1.2) and the highest cerium (K5.3) amounts. The regular distribution and defined morphology of cells for K5.3 confirm the excellent performances obtained with cellular tests (cellular vitality and proliferation).

Our previous studies have shown that in our glasses there is a simultaneous presence of Ce^3+^ and Ce^4+^ ions. [28] Furthermore, Naganuma and Traversa [50] found that cell proliferation and adhesion of cerium-doped materials are related to the cerium oxidation state (Ce^3+^ vs. Ce^4+^):Ce^3+^ ions inhibit cell proliferation and Ce^4+^ ions promote cell proliferation.

The glasses with a high cerium amount (K3.6 and K5.3) give rise to the formation of CePO_4_ on the surface; we can, thus, postulate that this phase can be competitive and slows down HA formation but at the same time stabilizes Ce^3+^ inhibiting its negative effects on cellular proliferation as confirmed from the cytocompatibility results. In fact, cytocompatibility results show that cellular viability increases with cerium amounts. The cellular vitality of K5.3 increases also with the dilution (1:2 and 1:5) after 24 h: therefore K5.3 glass at lower concentrations has again a good effect on cellular viability.

Furthermore, with respect to the previous (H-derived) studied glasses [36], the glasses investigated here show a higher cytocompatibility, as especially evident for cell proliferation that is greater than 100% for K5.3.

## 4. Conclusions

The elementary and mineralogical analyses indicate that all doped glasses are bioactive according to a bioactivity assessment based on European directives.

At high amounts of cerium (K3.6 and K5.3 glasses) HA formation is slower due to the competitive precipitation of CePO_4_; however, CePO_4_ reduces the negative effect of Ce^3+^ ions on cell proliferation by precipitating them as phosphates.

As a result, both vitality and cell proliferation are increased by using glasses with higher concentrations of cerium in them; both direct and indirect cytocompatibility tests show this trend. The results deriving from the contact between K3.6 and K5.3 and murine osteocyte (MLO-Y4) cell lines lead the way to the biological evaluation of these glasses also in vivo, to verify if the in vitro behavior is also emulated with an osteoregenerative action in a short time. However, the in vitro biological test has shown a clear positive action on cell proliferation, compared to glasses derived from H. These glasses, therefore, have the potential to provide a new class of biomaterials for hard tissue applications.

## Figures and Tables

**Table 1:** Nominal composition of the synthesized glasses (mol%).

Sample	SiO_2_	Na_2_O	CaO	CeO_2_
K	50.0	25.0	25.0	–
K1.2	49.4	24.7	24.7	1.2
K3.6	48.2	24.1	24.1	3.6
K5.3	47.3	23.7	23.7	5.3

**Table 2:** One-way analysis of variance results of cytotoxicity tests.

Tests	P	R^2^
NR after 24 h	<0.0001	0.7884
NR after 72 h	<0.0001	0.9779
MTT 24 h	0.1179	0.3087
MTT 72 h	0.0461	0.4174
BrdU 24 h	<0.0001	0.852
BrdU 72 h	<0.0001	0.7477

**Table 3:** Dunnett’s multiple comparison test results of cytotoxicity tests.

Tests	Dunnett’s Multiple Comparison Test *p* Value (≤0.05 Statistically Significant)			
	CTRL– vs K	CTRL– vs K 1.2	CTRL– vs K 3.6	CTRL– vs K 5.3
NR after 24 h	Yes	Yes	No	Yes
NR after 72 h	Yes	Yes	Yes	Yes
MTT after 24 h	No	No	No	No
MTT after 72 h	No	No	Yes	No
BrdU after 24 h	Yes	Yes	No	Yes
BrdU after 72 h	No	No	Yes	No
